# Recurrent Euglycemic Diabetic Ketoacidosis Weeks After SGLT2 Inhibitor Cessation: A Case Report

**DOI:** 10.1155/crie/9610048

**Published:** 2026-09-25

**Authors:** Celine Mansour, Emile Katrib, Myriam Mansour, Mirna Fares

**Affiliations:** ^1^ Faculty of Medicine and Medical Sciences, University of Balamand, Koura, Lebanon, balamand.edu.lb; ^2^ Faculty of Medicine, American University of Beirut, Beirut, Lebanon, aub.edu.lb; ^3^ Mount Lebanon Hospital, Hazmieh, Lebanon

**Keywords:** case report, critical illness, dapagliflozin, euglycemic diabetic ketoacidosis, sepsis, SGLT2 inhibitor

## Abstract

Euglycemic diabetic ketoacidosis (EDKA) is a complication of sodium‐glucose cotransporter‐2 (SGLT2) inhibitors that is being increasingly documented. It is challenging to diagnose, especially in critically ill patients because hyperglycemia is absent or mild. We report the case of a 71‐year‐old man with type 2 diabetes mellitus previously treated with dapagliflozin, which had been discontinued 2 weeks before presentation, who was admitted for tracheostomy and percutaneous endoscopic gastrostomy (PEG) placement in the context of a neurodegenerative disorder. On postoperative day 2, he was transferred to the intensive care unit (ICU) for aspiration pneumonia complicated by septic shock. He subsequently experienced recurrent episodes of high anion gap metabolic acidosis associated with positive ketonuria and relative euglycemia. The initial episode was interpreted as mixed septic lactic acidosis and SGLT2 inhibitor‐associated EDKA, whereas later episodes were more consistent with isolated recurrent EDKA. Management consisted of treatment of the underlying septic shock, and repeated courses of fixed intravenous (IV) insulin infusions to close the anion gap while maintaining euglycemia. This case demonstrates the importance of suspecting EDKA, especially in critically ill patients on SGLT2 inhibitors who present with unexplained high anion gap metabolic acidosis, even when lactic acidosis is present. Early diagnosis and treatment are essential to prevent complications and relapse.

## 1. Introduction

Sodium‐glucose cotransporter‐2 (SGLT2) inhibitors are considered second‐line treatment for type 2 diabetes mellitus, with additional indications for heart failure and chronic kidney disease due to their cardiorenal protective effects [1, 2]. Although these medications are widely used and their efficiency has been documented and supported by the literature, they carry a small but important risk of EDKA, characterized by ketonemia, metabolic acidosis, and a high anion gap despite normal to mildly elevated plasma glucose levels [3]. The overall prevalence of EDKA in patients on SGLT2 inhibitor drugs is relatively low [4]. However, the number of cases presenting as EDKA is increasing, probably due to the increased use of this drug class in the treatment of diabetes [3–5].

EDKA is often challenging to diagnose, especially in the intensive care unit (ICU), where high anion gap metabolic acidosis is frequently attributed to acute kidney injury or lactic acidosis secondary to septic shock and other causes of tissue hypoperfusion [6]. Sepsis, surgery, reduced oral intake, and reduced insulin doses are known risk factors for SGLT2‐associated EDKA [7, 8]. Perioperative and postprocedural cases demonstrate that EDKA can occur despite the discontinuation of SGLT2 inhibitors many days before the triggering event and that prolonged glucosuria and ketonuria might be observed [9, 10].

We present a complex case of an elderly man with septic shock following aspiration pneumonia and chronic dapagliflozin exposure, who developed recurrent SGLT2‐inhibitor‐associated EDKA. This case is noteworthy because it suggests that recurrent EDKA may occur weeks after SGLT2 inhibitor cessation in the presence of major precipitating stressors.

## 2. Case Presentation

A 71‐year‐old man with a history of type 2 diabetes mellitus, hypertension, dyslipidemia, benign prostatic hyperplasia, and a neurodegenerative disorder, as well as a past surgical history of transurethral resection of the prostate, cholecystectomy, and laminectomy, was admitted for tracheostomy and percutaneous endoscopic gastrostomy (PEG). The patient was on aspirin 100 mg daily, valsartan 160 mg daily, atorvastatin 10 mg daily, dapagliflozin 10 mg daily, and vildagliptin/metformin 1000 mg daily. Dapagliflozin was taken chronically by the patient for glycemic control and cardiorenal protection and was stopped 2 weeks prior to hospitalization.

On postoperative day 2, he was transferred to the ICU for aspiration pneumonia complicated by septic shock. Upon admission to the ICU, laboratory investigations showed leukocytosis with elevated inflammatory markers, including a white blood cell count of 17.5 × 10^3^/µL, C‐reactive protein of 131 mg/L, and procalcitonin of 0.289 ng/mL. Lactate was significantly elevated at 29.1 mg/dL (normal <20 mg/dL), consistent with severe septic lactic acidosis, and the anion gap was 22. Initial arterial blood gas demonstrated mild metabolic acidosis with a pH of 7.349 and a bicarbonate of 19.9 mmol/L. The patient was started on broad‐spectrum antibiotics and a norepinephrine infusion following adequate fluid resuscitation to maintain a mean arterial pressure (MAP) above 65 mmHg. The patient improved clinically and was successfully weaned to a very low dose of pressors within 24 h.

On the following day, despite hemodynamic stability, the patient developed a high‐anion gap metabolic acidosis with relative euglycemia. Arterial blood gas analysis showed severe acidemia with a pH of 7.178, a PCO_2_ of 33.0 mmHg, a bicarbonate of 11.8 mmol/L, and a normal level of lactic acid with an estimated anion gap of ~33. Urinalysis demonstrated glycosuria and marked ketonuria (3+). The patient was initiated on a fixed‐rate intravenous (IV) insulin infusion in conjunction with dextrose‐containing IV fluids to maintain euglycemia until resolution of the anion gap in line with EDKA management recommendations, demonstrating the superiority of fixed‐rate IV insulin and dextrose‐containing fluids to maintain euglycemia in the treatment of such cases [11]. IV insulin was discontinued within 48 h; however, it was reintroduced the following day due to the recurrence of metabolic acidosis. Subsequently, the patient was successfully transitioned from IV to subcutaneous insulin therapy, with normalization of acid–base status (pH 7.479, serum bicarbonate 29.0 mmol/L) and restoration of a normal anion gap.

Enteral nutrition was provided through PEG as a gavage feeding of 350 mL every 6 h, with a nutritional target of more than 1500 kcal/day. However, this target was not consistently achieved on a daily basis during the patient’s critical illness, resulting in periods of reduced caloric intake that may have contributed to the ketogenic state. Following discontinuation of the first IV insulin course, the patient was subsequently managed with subcutaneous rapid‐acting insulin (Actrapid) according to bedside glucose measurements. Because the available records document the prescribed protocols rather than complete cumulative insulin and dextrose doses, exact total doses are not reported.

Two weeks following the initial episode of EDKA, the patient experienced a recurrent severe acidotic event, with arterial blood gas analysis demonstrating a pH of 7.201, a PCO_2_ of 29.4 mmHg, and a serum bicarbonate of 11.1 mmol/L, accompanied by ketonuria. The EDKA management protocol was reinitiated, leading to the subsequent correction of the anion gap and metabolic derangements.

After reinitiation of IV insulin, the metabolic abnormalities again resolved, with subsequent near‐normal arterial blood gases and closure of the anion gap. Dapagliflozin was not restarted during the documented hospitalization. During the available postdischarge follow‐up period, no further episodes of EDKA or recurrent ketoacidosis were documented.

Throughout these episodes of EDKA, urinary ketones were persistently positive in association with glycosuria despite the absence of marked hyperglycemia. Serial bedside capillary blood glucose measurements (HGT) demonstrated euglycemic to only mildly elevated values, without any documented episodes of significant hyperglycemia, as typically observed in classic diabetic ketoacidosis. Representative glucose ranges were 113–200 mg/dL between February 22–24, 88–156 mg/dL between February 26 and March 1, and 108–200 mg/dL between March 7–11. These findings met the biochemical criteria for EDKA, defined by a blood glucose level <200–250 mg/dL in the presence of ketonemia/ketonuria and high anion gap metabolic acidosis. The initial acid–base disturbance was interpreted as a mixed high anion gap metabolic acidosis, with septic lactic acidosis considered the primary driver and concomitant SGLT‐2 inhibitor‐associated euglycemic diabetic ketoacidosis (EDKA) as a contributing factor. In contrast, the subsequent episodes of acid–base derangement were attributed exclusively to EDKA.

**Figure 1:**
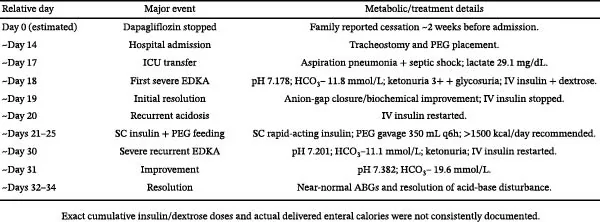
Clinical and metabolic timeline. Day 0 represents the estimated date of dapagliflozin discontinuation, ~14 days before admission based on family report. Relative days are used to protect patient confidentiality.

The temporal relationship between dapagliflozin discontinuation, the patient’s clinical course, recurrent episodes of metabolic acidosis, and insulin treatment is summarized in Table 1 and illustrated in Figure 1.

**Table 1:** Summary of key events and findings.

Relative day/period	Event and findings
~Day 14	Hospital admission for tracheostomy and PEG placement; dapagliflozin had reportedly been stopped ~2 weeks earlier.
~Day 17	ICU admission for aspiration pneumonia and septic shock; lactate 29.1 mg/dL; initial HAGMA.
~Day 18	Severe HAGMA (pH 7.178, HCO_3_‐ 11.8 mmol/L), marked ketonuria with glycosuria and normal lactate; IV insulin with dextrose‐containing fluids initiated.
~Day 19	Biochemical improvement and anion‐gap closure; IV insulin discontinued.
~Day 20	Recurrent metabolic acidosis; IV insulin reinitiated.
~Days 21–25	Predominantly euglycemic HGT; transition to subcutaneous rapid‐acting insulin. PEG gavage feeding 350 mL q6h prescribed; nutritional target >1500 kcal/day, but target caloric intake was not consistently achieved.
~Day 30	Recurrent severe acidosis (pH 7.201, HCO_3_‐ 11.1 mmol/L) with ketonuria; IV insulin restarted.
~Day 31	Improving metabolic status (pH 7.382, HCO_3_‐ 19.6 mmol/L).
~Days 32–34	Near‐normal ABGs and resolution of acid–base disturbance.

Abbreviations: EDKA, euglycemic diabetic ketoacidosis; HAGMA, high anion gap metabolic acidosis; ICU, intensive care unit.

## 3. Discussion

The present report describes a case of EDKA that initially developed ~2 weeks after discontinuation of SGLT2 inhibitors and subsequently recurred in a severe form 4 weeks after cessation of therapy. SGLT2 inhibitors might cause glycosuria, relative insulinopenia, and increased glucagon, which predisposes to ketogenesis even with normal plasma glucose levels, especially in the context of catabolic stressors like infection, decreased nutrition, surgery, and critical illness [12, 13]. In this patient, EDKA was precipitated by a combination of factors, including aspiration pneumonia, systemic inflammation, recent surgery, reduced nutritional intake following newly initiated PEG feeding, and chronic use of dapagliflozin. Chow et al. [5] determined diagnostic criteria for EDKA as blood glucose <200 mg/dL, serum β‐hydroxybutyrate ≥3.0 mmol/L, and at least one of arterial pH ≤7.3, bicarbonate ≤18 mEq/L, or anion gap >10. Although serum β‐hydroxybutyrate was not documented in this case, the presence of severe acidemia, decreased bicarbonate levels, elevated anion gap, persistent ketonuria, and relative euglycemia aligns with this EDKA definition. Recent reviews have mentioned that EDKA might be considered with glucose up to 250–300 mg/dL in practice, which supports the idea that a strict numeric threshold cannot exclude the diagnosis of EDKA when the clinical context and acid–base profile are suggestive of it [5, 7, 14].

Although dapagliflozin has a plasma elimination half‐life of ~12–13 h, the plasma half‐life may not fully reflect the duration of its pharmacodynamic effects. Previous reports have described SGLT2 inhibitor‐associated EDKA occurring 1–2 weeks after treatment cessation, persistent glycosuria for several days after discontinuation, and recurrence of ketoacidosis after apparent metabolic improvement. Bobrowski et al. [15] reported persistent glycosuria and ongoing SGLT2 inhibitor‐associated pharmacologic effects up to 14 days after discontinuation, substantially exceeding the expected drug clearance period of approximately five half‐lives. The mechanism underlying these prolonged effects remains incompletely understood. These observations suggest that elimination half‐life alone may not reliably define the period during which SGLT2 inhibitor‐associated metabolic effects remain clinically relevant. In our patient, dapagliflozin had reportedly been discontinued approximately 2 weeks before the admission, and recurrent episodes of ketoacidosis subsequently occurred during hospitalization. Although persistence of dapagliflozin itself cannot explain such a prolonged interval and drug concentrations were not measured, previously reported prolonged pharmacodynamic effects, together with ongoing ketogenic stressors including insufficient caloric intake, sepsis, surgery, critical illness, and relative insulin deficiency, may have contributed to the delayed and recurrent presentation.

Prior exposure to SGLT2 inhibitors likely created a prolonged ketogenic milieu that increased susceptibility to EDKA, whereas concomitant sepsis, surgery, reduced nutritional intake, critical illness, and altered metabolic demands acted as precipitating factors. The recurrent episodes of high‐anion gap metabolic acidosis and ketonuria in the setting of relative euglycemia strongly supported recurrent EDKA as the predominant mechanism underlying these events. A reduced caloric intake likely contributed to ketogenesis in this patient. Although enteral nutrition through PEG was prescribed at 350 mL every 6 h, with a nutritional target exceeding 1500 kcal/day, the patient’s actual caloric intake remained insufficient throughout the clinical course. Enteral feeding had to be interrupted during surgical procedures and periods of clinical instability, particularly during septic episodes, and was subsequently resumed gradually as tolerated. Thus, prolonged and intermittent underfeeding, in addition to the metabolic effects associated with recent SGLT2 inhibitor exposure, may have contributed to the development and recurrence of ketoacidosis.

Recent literature increasingly reports cases of SGLT2 inhibitor‐associated EDKA precipitated by sepsis, including instances involving empagliflozin, in which diagnosis was delayed until persistent high‐anion gap metabolic acidosis and ketonemia were recognized [16].

Guidelines suggest that SGLT2 inhibitors must be withheld for at least 3–4 days before elective surgery and during any acute illness, dehydration, or reduced oral intake because of the risk of EDKA [17]. Furthermore, recent data suggest that restarting SGLT2 inhibitors soon after the resolution of an EDKA episode can be associated with recurrent DKA or symptomatic ketosis and that the reinitiation of the medication must be individualized, taking into consideration the risks and the benefits. In ICU patients with recurrent acidosis and associated comorbidities, permanent discontinuation should be considered [5].

This case also highlights the risk of missing the diagnosis of EDKA. In the setting of septic shock, elevated lactate levels may mislead clinicians to attribute the metabolic acidosis solely to lactic acidosis, thereby overlooking concomitant ketoacidosis, particularly when blood glucose levels are only mildly elevated or within the normal range. Several case reports have emphasized that EDKA associated with SGLT2 use is frequently under‐recognized and that clinicians must always suspect it in patients on SGLT2 with unexplained persistent high anion gap acidosis, especially with ketonuria or ketonemia [5, 6, 11, 18, 19]. In the present case, the diagnosis of SGLT2 inhibitor‐associated EDKA is supported by the characteristic combination of high anion gap metabolic acidosis, marked ketonuria, glycosuria, normal lactate levels, and relative euglycemia, together with recurrent episodes and subsequent improvement following insulin‐based EDKA treatment. Nevertheless, because serum beta‐hydroxybutyrate was not measured and several concurrent ketogenic stressors were present, a direct causal relationship with dapagliflozin cannot be definitively established. Accordingly, the term “SGLT2 inhibitor‐associated EDKA” is used to reflect the likely contribution of dapagliflozin without implying that it was the sole precipitating factor.

**Figure 2:**
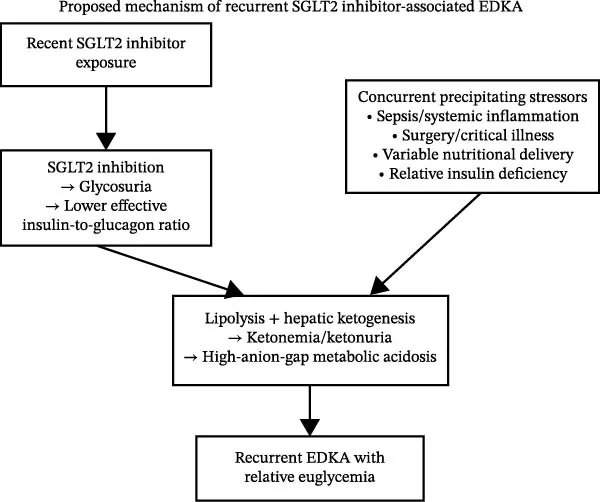
Proposed pathophysiological framework for recurrent SGLT2 inhibitor‐associated EDKA. Recent SGLT2 inhibitor‐associated metabolic effects are distinguished from ongoing precipitating stressors. Late recurrence does not imply persistence of dapagliflozin itself; ongoing metabolic stress may sustain susceptibility to ketogenesis after drug elimination.

The management of SGLT2‐associated EDKA is similar to that of classic DKA; however, it requires some modifications. Because glucose levels are usually normal or mildly elevated, variable‐rate insulin infusions cannot be used alone. Instead, fixed‐rate IV insulin must be administered with dextrose‐containing fluids to avoid hypoglycemia while the anion gap returns gradually to normal. Chow et al. [5] recommend continuing insulin until the normalization of anion gap and ketone levels. Afterward, the transition to subcutaneous insulin must be done cautiously with at least 24 h of strict observation to prevent relapse [5]. The proposed interaction between recent SGLT2 inhibitor exposure and the patient’s concurrent precipitating factors is summarized in Figure 2.

This report has several limitations. Serum beta‐hydroxybutyrate was not measured because this assay was not available at our institution, the exact date of dapagliflozin cessation was unavailable and was estimated from the family report, and complete cumulative IV insulin and dextrose administration, as well as actually delivered enteral calorie data, were not consistently documented. These limitations prevent the definitive attribution of every episode to dapagliflozin alone. Nevertheless, the serial acid–base profile, marked ketonuria, glycosuria, relative euglycemia, normal lactate level, and response to insulin‐based therapy support the diagnosis of recurrent SGLT2 inhibitor‐associated euglycemic diabetic ketoacidosis (EDKA).

## 4. Conclusion

In critically ill patients with type 2 diabetes who present with high anion gap metabolic acidosis, positive ketones, and only modest hyperglycemia, SGLT2 inhibitor‐associated EDKA should be considered, even when the SGLT2 inhibitor has already been discontinued. Although dapagliflozin has a relatively short plasma elimination half‐life, previously reported pharmacodynamic effects of SGLT2 inhibition may persist beyond the expected drug clearance period, including beyond five elimination half‐lives. Clinicians should therefore maintain a high index of suspicion for EDKA following recent SGLT2 inhibitor discontinuation, particularly when ongoing precipitating factors are present. Late or recurrent EDKA should not be interpreted as evidence of persistence of dapagliflozin itself but may reflect prolonged metabolic susceptibility to ketogenesis in the setting of acute illness, surgery, insufficient caloric intake, and relative insulin deficiency. Recognition and correction of these concurrent ketogenic stressors, together with appropriate EDKA‐directed therapy, are essential to reduce the risk of recurrence.

**Nomenclature**
ABG: Arterial blood gas
DKA: Diabetic ketoacidosis
EDKA: Euglycemic diabetic ketoacidosis
HAGMA: High anion gap metabolic acidosis
HGT: Hemoglucotest (capillary blood glucose measurement)
ICU: Intensive care unit
IV: Intravenous
MAP: Mean arterial pressure
PCO_2_: Partial pressure of carbon dioxide
PEG: Percutaneous endoscopic gastrostomy
SGLT2: Sodium‐glucose cotransporter‐2.

## Author Contributions

Celine Mansour and Emile Katrib were responsible for data collection, literature review, manuscript drafting, and interpretation of clinical data. Mirna Fares supervised the patient’s clinical management and contributed to the critical revision of the manuscript. Myriam Mansour contributed to the literature review, manuscript editing, formatting, and coordination of submission.

## Funding

The authors received no specific funding for this work.

## Disclosure

All authors read and approved the final manuscript. Mirna Fares had full access to all of the data in this study and takes complete responsibility for the integrity of the data and the accuracy of the data analysis. All AI‐assisted content was critically reviewed and verified by the authors.

## Ethics Statement

The authors have nothing to report.

## Consent

Written informed consent for the publication of this case report was obtained from the patient.

## Conflicts of Interest

The authors declare no conflicts of interest.

## Data Availability

The data that support the findings of this study are available from the corresponding author upon reasonable request.
